# Task Cortical Connectivity Reveals Different Network Reorganizations between Mild Stroke Patients with Cortical and Subcortical Lesions

**DOI:** 10.3390/brainsci13081143

**Published:** 2023-07-29

**Authors:** Jiaye Cai, Mengru Xu, Huaying Cai, Yun Jiang, Xu Zheng, Hongru Sun, Yu Sun, Yi Sun

**Affiliations:** 1 Department of Neurology, Sir Run Run Shaw Hospital, Zhejiang University School of Medicine, Hangzhou 310020, China; caijiaye@zju.edu.cn (J.C.); caihuaying2004@zju.edu.cn (H.C.); jiangyunmail@zju.edu.cn (Y.J.); hsy033@163.com (X.Z.); yusun@zju.edu.cn (Y.S.); 2 Key Laboratory for Biomedical Engineering of Ministry of Education, Department of Biomedical Engineering, Zhejiang University, Hangzhou 310027, China; 3 Department of Electrocardiogram, Dongyang Traditional Chinese Medicine Hospital, Dongyang 322100, China; sunhr0621@163.com; 4 MOE Frontiers Science Center for Brain Science and Brain-Machine Integration, Zhejiang University, Hangzhou 310058, China; 5 State Key Laboratory for Brain-Computer Intelligence, Zhejiang University, Hangzhou 310016, China

**Keywords:** EEG source localization, power envelope connectivity, graph theory, cognitive impairment, mild stroke

## Abstract

Accumulating efforts have been made to investigate cognitive impairment in stroke patients, but little has been focused on mild stroke. Research on the impact of mild stroke and different lesion locations on cognitive impairment is still limited. To investigate the underlying mechanisms of cognitive dysfunction in mild stroke at different lesion locations, electroencephalograms (EEGs) were recorded in three groups (40 patients with cortical stroke (CS), 40 patients with subcortical stroke (SS), and 40 healthy controls (HC)) during a visual oddball task. Power envelope connectivity (PEC) was constructed based on EEG source signals, followed by graph theory analysis to quantitatively assess functional brain network properties. A classification framework was further applied to explore the feasibility of PEC in the identification of mild stroke. The results showed worse behavioral performance in the patient groups, and PECs with significant differences among three groups showed complex distribution patterns in frequency bands and the cortex. In the delta band, the global efficiency was significantly higher in HC than in CS (*p* = 0.011), while local efficiency was significantly increased in SS than in CS (*p* = 0.038). In the beta band, the small-worldness was significantly increased in HC compared to CS (*p* = 0.004). Moreover, the satisfactory classification results (76.25% in HC vs. CS, and 80.00% in HC vs. SS) validate the potential of PECs as a biomarker in the detection of mild stroke. Our findings offer some new quantitative insights into the complex mechanisms of cognitive impairment in mild stroke at different lesion locations, which may facilitate post-stroke cognitive rehabilitation.

## 1. Introduction

Stroke has become the leading cause of disability and death in China, with more than 2 million patients suffering from stroke each year [1]. Notably, cognitive impairments are common after stroke, and their combined impact substantially increases the cost of care and healthcare costs [2]. Consequently, a growing body of research investigates the neural mechanisms and functional rehabilitation of cognitive dysfunction after stroke [3,4]. However, most studies to date have focused on moderate to severe stroke patients [5,6], while patients with mild stroke have received less attention. According to the National Institutes of Health Stroke Scale (NIHSS), mild stroke is defined as NIHSS < 5 [7].  Cognitive impairments, including executive function and attention, are found in mild stroke, although the deficits are subtle in comparison to the more obvious symptoms of moderate to severe stroke (e.g., hemiparesis and aphasia) [8]. Previous longitudinal studies demonstrated that even in the chronic phase of mild stroke, cognitive dysfunction persists and affects the daily life of mild stroke patients [9,10]. Given that mild stroke accounts for a significant proportion of post-stroke patients [11], it is necessary to further explore the cognitive impairments and the underlying mechanisms induced by mild stroke.

Cognitive dysfunction has been demonstrated to be related to lesion location [12]. However, there is limited and inconsistent evidence on the effect of different lesion locations on cognitive impairment. For example, neuropsychological tests have shown that patients with acute subcortical lesions have significantly worse cognitive function, including attention and memory, than those with acute cortical lesions [13]. Clinically, it is generally believed that cortical damage is more severe than subcortical damage, even though basal ganglia strokes can affect the performance of any cognitive function [14]. However, a comprehensive neuropsychological assessment, focusing specifically on executive and attentional functions, found no significant differences between acute cortical and subcortical stroke patients [15]. Of note, these studies did not include mild stroke. Mild stroke patients often exhibit cognitive dysfunction, and the effect of lesion location on cognitive deficits remains unclear. Therefore, extending the investigation to mild stroke and further including patients with different lesion locations may help to understand the neurological mechanisms of lesion location-dependent cognitive impairment in mild stroke patients.

Electroencephalogram (EEG), as a non-invasive neuroimaging technique, has been widely employed to analyze the functional pathophysiology of brain disorders and neuromechanisms in cognition [16,17]. Essentially, the structural lesion caused by a focal brain lesion has a profound impact on the topology of the entire functional brain network, altering the connectivity of the lesion and remote regions from the lesion [18]. Recently, EEG functional connectivity features were found to be associated with lesion location in a study that focused on patients with different lesion locations [19]. Moreover, by assessing resting-state EEG functional connectivity, Dubovik et al. found a unique correlation of cognitive function with alpha-band functional connectivity in post-stroke patients [20]. Although EEG has advantages, such as high temporal resolution, good clinical practicality, convenience, and low cost, there is a challenge that needs to be overcome. Specifically, the issue of volume conduction can lead to confusion in determining whether scalp EEG channels are detecting unique or shared sources due to the attenuation and dispersion of neural sources as they reach the scalp [21]. This limitation in spatial resolution can result in the inaccurate determination of the location of the neural source. To overcome this challenge, power envelope connectivity (PEC) was recently introduced, effectively mitigating the impact of volume conduction and providing accurate estimates of the associated structure of EEG spontaneous oscillatory activity [22]. PEC estimation uses orthogonalization to remove signals with zero phase lag to reduce spurious correlations due to volume conduction [23]. It differs from connectivity measures based on coherence and phase, which rely on correlations between the instantaneous amplitudes of oscillatory signals across regions (called power envelopes). Notably, PEC has been used to delineate the connectomic profile of disorders and identify disease subtypes with satisfactory results [23,24], demonstrating the validity and reliability of this method in connectivity construction.

Graph theory analysis (GTA) has been applied to the analysis of functional brain networks due to its ability to evaluate the topological properties of the network [25]. Graph measures characterize local and global network properties, including the degree of local connectedness of the network (e.g., clustering coefficient (CC) and local efficiency ($E_{local}$)) and the ability to transmit information throughout the entire network (e.g., characteristic path length (*L*) and global efficiency ($E_{global}$)) [25,26]. In addition, small-world networks are characterized by high CC and small *L*, which measure the ability of the brain networks to process the flow of information both locally and globally [27]. By calculating the small-worldness (σ) of resting-state EEG signals, Caliandro and colleagues found that network rearrangements in stroke patients were found in delta, theta, and alpha bands [18]. Additionally, a study focusing on the cognitive process in stroke patients showed longer *L* and decreased CC in the mental rotation process, suggesting impaired segregation and integration [28]. The study of the impact of focal damage on functional brain networks using GTA not only reveals stroke-related changes from the local to the global scale [29,30] but also promotes the investigation of topological changes and neural communication during cognitive tasks [28]. Of note, it is widely accepted that executive control and attention are the cognitive processes most commonly impaired in stroke patients [31], and attention is critical for several cognitive functions, including visual selection and attentional allocation [32]. Given that the visual oddball paradigm adopted in this work has widely been used to investigate the mechanisms of attention and executive functions [33,34], and that mild stroke is usually accompanied with cognitive deficits [35], we, therefore, hypothesize that cognitive impairment would be demonstrated through task performance and the altered functional network topology of the brain. Furthermore, the effect of different lesion locations on cognitive function remains unclear [14,15]. Thus, we propose a second hypothesis that functional brain network structures differ in cortical and subcortical stroke patients during cognitive processes. In addition, to explore the feasibility of PEC as a biomarker, we further attempted to identify patients with mild stroke based on PEC characteristics. In order to investigate the discriminative ability of PEC in a global manner (i.e., whether satisfactory classification accuracy was achieved despite the different feature selection (FS) methods and classifiers employed), several common FS methods and classifiers validated in previous studies for brain disease classification were used in this study [36,37,38]. To this end, EEG data were recorded during the visual oddball task in three groups (i.e., patients with cortical stroke, patients with subcortical stroke, and healthy controls). Specifically, to determine the effect of lesion location, we divided patients into two groups (a cortical (CS) lesion group and a subcortical (SS) lesion group). Brain lesions assessed by computed tomography (CT)/magnetic resonance imaging (MRI) were defined as CS if they involved the frontal, temporal, parietal, and occipital lobes, and as SS if the lesions involved the subcortical regions, including the internal capsule, basal ganglia, thalamus, and cerebellum. We first constructed PEC based on the source-space EEG signals obtained by performing source localization. Subsequently, changes in the functional brain network properties were quantitatively assessed using GTA. A further exploratory classification analysis was conducted on patients using PEC as a feature to explore the potential of PEC as a biomarker.

## 2. Materials and Methods

### 2.1. Participants

Forty CS patients and forty SS patients with mild stroke were recruited during annual routine health check-ups at the Department of Neurology, Sir Run Run Shaw Hospital, Zhejiang University School of Medicine. Inclusion criteria for patients with mild stroke included (1) age > 18 years, (2) diagnosed with mild stroke symptoms based on the NIHSS with a score < 5), (3) no neurological or psychiatric comorbidity, (4) reported no visual impairment, and (5) able to understand task instructions and perform the task independently. Forty age- and sex-matched healthy controls (HCs) were recruited through advertisement as a control group. All of the HCs reported having no history of psychiatric or neurological conditions. All participants in the current study had a normal or corrected-to-normal vision and were right handed. The specific forms of rehabilitation performed on the patients recruited in the current work are listed in Table A1. Table 1 shows the characteristics of the three groups. The experimental protocol was approved by the Institutional Review Boards of Sir Run Run Shaw Hospital, Zhejiang University School of Medicine (protocol code 20200115-33, 15 January 2020). Each participant in the study provided written informed consent according to the Declaration of Helsinki.

### 2.2. Experimental Paradigms

**Figure 1:**
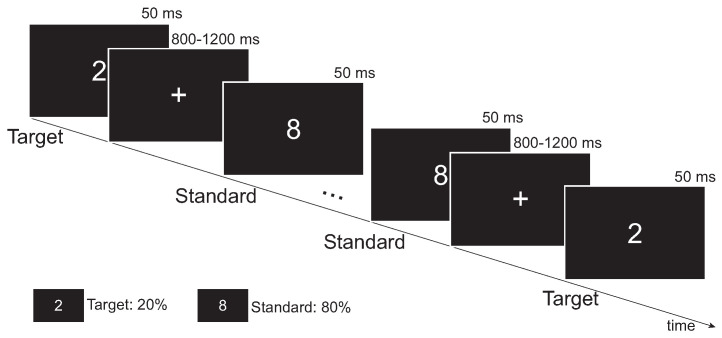
Experimental paradigm of the visual oddball task.

To investigate mild stroke-induced functional brain network changes in cognitive processing, the visual oddball task, a previously validated paradigm involving executive and attentional functions [33,34], was adopted in this study. Specifically, participants sat in a soundproof room, facing a computer monitor. Figure 1 illustrates the visual oddball task paradigm. The visual stimuli were presented through the software E-prime. Infrequent target stimuli (white number 2) were presented randomly with frequent standard stimuli (white number 8). The probabilities of target and standard stimuli were 20% and 80%, respectively. The presentation of visual stimuli lasted for 50 ms. The inter-stimulus interval (ISI) ranged from 800 to 1200 ms. In total, 500 visual stimuli were presented. The task took approximately 9 min. Participants were required to respond by pressing the response button (the leftmost button on the response pad) as soon as the target stimulus was presented.

### 2.3. EEG Recordings and Preprocessing

EEG signals were acquired during the task at a sampling rate of 1000 Hz according to the international 10–20 system using a 60-channel NeuroScan SynAmps2 Amplifier. Electrodes placed lateral to the external canthus and above/below the orbit of the left eye were used to record horizontal and vertical electrooculogram (EOG) signals. In the Quik-Cap supplied with the SynAmps2, the electrode placed between the CPZ and CZ was used as the reference electrode. During data collection, the impedance of the electrodes was kept below 5 kΩ, and a 50 Hz notch filter was employed to minimize interference. A previously validated pre-processing procedure was employed [39]. Raw EEG signals were downsampled to 256 Hz and bandpass filtered (1–40 Hz), and the average was re-referenced. Bad epochs were then removed by visual inspection. The mean ± SEM numbers of rejected epochs were 4.73 ± 1.55, 6.43 ± 1.68, and 8.90 ± 2.11 for the HC, CS, and SS groups, respectively, with no significant group difference ($F_{2,117}$ = 1.364, *p* = 0.260). Afterwards, independent component analysis (ICA) was employed to eliminate artifacts resulting from muscle activity, as well as ocular artifacts by removing components with strong correlations to the EOG signals. The mean ± SEM numbers of rejected ICA components were 6.05 ± 0.56 in the HC group, 4.45 ± 0.60 in the CS group, and 4.92 ± 0.43 in the SS group, with no significant group difference ($F_{2,117}$ = 2.368, *p* = 0.098). EEG data were then filtered into four canonical frequency bands: delta (1–4 Hz), theta (4–7 Hz), alpha (8–12 Hz), and beta (13–30 Hz). Then, they were segmented into [−100, 500] ms, with 0 ms indicating the target stimulus onset. Baseline correction was performed using [−100, 0] ms data. The preprocessing of the EEG data was carried out with custom scripts and the EEGLAB toolbox [40] in MATLAB 2021a (The MathWorks Inc., Natick, MA, USA).

### 2.4. Source-Space PEC Calculation

**Figure 2:**
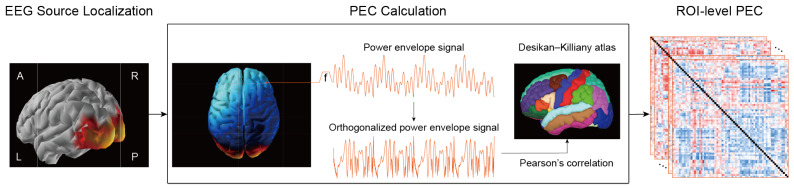
A framework for source-space PEC estimation. EEG source localization was implemented using weighted minimum norm estimation (wMNE) to transform sensor space EEG signals into source-space signals. The power envelope of each vertex was computed from the analytic signal derived from the Hilbert transform and orthogonalized for all the other vertices. The PEC was calculated as Pearson’s correlation coefficient between the power envelopes of each vertex pair. To extract ROI-level PEC values, 68 cortical regions were selected based on Desikan–Killiany atlas segmentation, and PEC values were averaged across all corresponding vertex pairs in these regions.

In order to delineate the functional brain network topology during cognitive processing and investigate the potential of functional connectivity as a feature for the identification of mild stroke, the source-space PEC of EEG signals was estimated. Figure 2 illustrates the overall framework of PEC construction.

#### 2.4.1. EEG Source Reconstruction

To minimize the influence of volume conduction, the source imaging approach was applied to transform the EEG signals into source-space signals [41]. In this study, source reconstruction was carried out according to [24]. Briefly, the three-layer head model was obtained based on the standard anatomical template ICBM152 [42]. The lead-field matrix was calculated using the boundary element method (BEM) [43] with the OpenMEEG plugin from BrainStorm toolbox [44]. The weighted minimum norm estimate (wMNE) was employed to estimate the free-oriented (3 orthogonal components for each dipole) source activities, and the regularization parameter was set to δ=1/100. Then the Euclidean norm of three-dimension source signals at each dipole was calculated to obtain the one-dimension time series for the following analysis.

#### 2.4.2. EEG Functional Connectivity Measurement

A Hilbert transform was utilized to generate the source-space analytical time series for each frequency band [23]. To eliminate spurious correlations introduced by limited spatial resolution, the analytical signal of each vertex was orthogonalized to that of all other vertices, followed by the calculation of their power envelopes [22]. In particular, at the sampling point *t*, the orthogonal component of the analytic signal Y(t) relative to the analytic signal X(t) was defined as

(1) $$Y_{⊥}\left(t\right)=\mathrm{imag}\left(Y\left(t\right)\frac{X{\left(t\right)}^{*}}{\left|X(t)\right|}\right),$$

with $X{\left(t\right)}^{*}$ being the conjugate of X(t). To eliminate zero-phase lag signals, the orthogonal components of each signal pair were obtained after orthogonalization. The power envelopes were then calculated by squaring the orthogonalized analytical signals and applying a logarithmic transformation to improve the normality of the power statistics [22]. Pearson’s correlation coefficient was used to estimate the PEC at each vertex pair, which was further extracted in 68 regions of interest (ROI) defined according to the Desikan–Killiany (DK) atlas parcellation [45]. For each pair of regions, PEC values were averaged across all possible vertex pairs. Thus, the number of unique regional pairwise connectivity features was 68×(68−1)/2=2278 for each frequency band. Estimation of the PEC was implemented with custom scripts written in MATLAB 2021a.

### 2.5. Network Analysis

To explore the impact of different lesion locations on functional brain networks, a GTA was employed to quantitatively estimate the network properties. Notably, weak and non-significant connections in a brain network may indicate that there are spurious connections present, which can potentially mask the topology of strong and significant connections [26]. Prior to the following GTA, to retain significant interactions, the widely used thresholding method was adopted, defining the threshold as the top 10% connections [46,47,48]. Therefore, the original PEC matrix was transformed into a binary connectivity matrix. The current work estimated five well-established graph metrics (i.e., CC, *L*, σ, $E_{global}$, and $E_{local}$). A network is defined as *G*, containing *N* nodes (N=68 in the current work), and CC is a measure of the number of triangles in the graph, which quantifies how well individual nodes are embedded in their local neighborhood. To evaluate the functional segregation of the network, a global measure called CC was computed as follows:

(2) $$CC=\frac{1}{N}\sum_{k\in G}\frac{1}{N_{G_{k}}\left(N_{G_{k}}-1\right)}\sum_{i,j\in G_{k}}a_{ij}a_{ik}a_{jk},$$

where $a_{ij}$ means the edge value between node *i* and *j*. *L* quantifies the network’s overall routing efficiency, which is a global measure of network functional integration, calculated as follows:

(3) $$L=\frac{1}{N(N-1)}\sum_{i\neq j\in G}L_{i,j},$$

where $L_{i,j}$ indicates the shortest distance between node *i* and *j*. Small-worldness σ is a measure for quantitatively evaluating small-world properties, which is defined as

(4) $$\sigma =\frac{CC/CC_{\mathrm{rand}\;}}{L/L_{\mathrm{rand}\;}},$$

where $CC_{rand}$ and $L_{rand}$ indicate the mean CC and mean *L* derived from 100 matched random networks, which are obtained by randomly reshuffling the edge while preserving the degree distribution and connectedness.

Since the small-world topology, characterized by higher CC compared to the random network and higher or similar *L* compared to the random network, is deemed to be optimal for the synchronization of neural activity between different brain regions [49], $E_{global}$ and $E_{local}$ were evaluated to give a direct and explicit physical interpretation for the notion of small-world properties from an information flow perspective. Specifically, $E_{global}$ quantifies the overall efficiency of the parallel information exchange throughout the entire network, which is inversely proportional to the *L*. $E_{global}$ is computed as

(5) $$E_{global}=\frac{1}{N(N-1)}\sum_{i\neq j\in G}\frac{1}{L_{i,j}}.$$

$E_{local}$ assesses the average efficiency of information transfer within subgraphs, calculated as

(6) $$E_{local}=\frac{1}{N}\sum_{i\in G}E_{global}\left(G_{i}\right),$$

where $E_{global}\left(G_{i}\right)$ denotes the global efficiency of the subgraph $G_{i}$, which is composed of the neighbors of the node *i*. The GTA in the current study was assessed by means of the Brain Connectivity Toolbox (BCT) [26] in MATLAB 2021a.

### 2.6. Feature Selection and Classifier

In order to further explore the feasibility of using PECs as features for the identification of mild stroke, a data-driven analysis was performed in this work. Different from hypothesis-driven statistical comparison methods, a classification framework based on data-driven analysis can eliminate the family-wise error rate, which often occurs in mass univariate analysis [50]. Data-driven analysis was performed for the classification of HC and CS, and HC and SS, respectively. Specifically, to explore the feasibility of PEC as a feature for identifying mild stroke from a more universal perspective, several widely used feature selection (FS) methods and classifiers were used in this work. The FS methods include correlation coefficients (Corr), the Fisher score algorithm (Fisher), relief, and least angle regression (LARS). The classifiers include the logistic regression model (LR), AdaBoost (Boost), decision tree (Tree), and random forest (RF). The selected FS methods and classifiers were validated in previous studies using functional connectivity as features for brain disease classification [36,37,38]. For those interested in the detailed description of the methods and classifiers, several recent reviews are available [51,52]. To evaluate the classification performance, leave-one (subject)-out cross validation (LOOCV) was employed in this study. In each iteration, all subjects were randomly assigned to either a training set or a test set, while the FS procedure was performed only on the training data, without using information from the test set, to avoid introducing bias. The training data set was used to build the model, while the accuracy was estimated on the test set [53]. The classification framework is shown in Algorithm 1.
**Algorithm 1:** Classification framework.**Input:** PEC features: $PEC\in R^{N\times F}$ Subject Index: $Subject\in R^{N},\;Subject_{i}\in \left\{1,2,\ldots N\right\}$ Labels of health/patient: $Label\in R^{N},Label_{i}\in \left\{1,2\right\}$**Output:** Classification accuracy: $ACC_{CV}$ The selected features: OptimalFeatures**Begin:** FeatureIndex=∅ **for** $\;Subject_{i}=1:N$ **do**   $PEC_{test}=PEC_{Subject_{i}}$   $PEC_{train}=PEC-PEC_{test}$   rank = FS$\left(PEC_{train},Label\right)$   **for** j=1:F **do**      selected=rank(1:j)      $D_{train}=PEC_{train}\left(:,selected\right)$      $D_{test}=PEC_{test}\left(:,selected\right)$      $model=train(D_{train},Label)$      $acc(Subject_{i},j)$$=classify(model,D_{test},Label)$      $FeatureIndex(Subject_{i},j)=selected$   **end for** **end for** $ACC_{CV}=mean\left(acc,1\right)\in R^{1\times F}$ Optimal number of features: $K=argmax(ACC_{CV})$ Optimal feature sets: Fsets=FeatureIndex(:,K) Rank features by occurrence rate:   
$rank_{o}=frequency\left(Fsets\right)$ Get index of K optimal features:   
$fIdx=Fsets(rank_{o}\left(1:K\right))$ OptimalFeatures=FC(:,fIdx)**End**

### 2.7. Statistical Analysis

Prior to the statistical analysis, the normality of all variables was evaluated by means of the Shapiro–Wilk test. This was followed by an estimation of homogeneity using Levene’s test. In the following analysis, parametric or non-parametric statistical methods were employed depending on the normality results. The statistical differences in the behavioral performance among the three groups were analyzed utilizing the Kruskal–Wallis test. To further compare these behavioral measures between groups, multiple comparison post hoc analysis with Bonferroni correction was performed. One-way ANOVA was employed to analyze the difference in the PECs across the three groups in each frequency band. The post hoc analysis was performed by the Bonferroni method in multiple comparisons of PECs between each two groups, and all *p*-values were multiple corrected. To reveal the differences in network metrics (i.e., CC, *L*, σ, $E_{global}$, and $E_{local}$), the Kruskal–Wallis test was applied. Then the Bonferroni post hoc test was utilized to identify specific differences. In this study, the statistical significance level was set to less than 0.05 (*p* < 0.05). Statistical analysis was executed with the use of SPSS 26 software (IBM, New York, NY, USA).

## 3. Results

### 3.1. Visual Task Results

The statistical comparison of behavioral performance, including the response accuracy (RA) and reaction time (RT), among the three groups is shown in Table 2. The relatively high RA in both patient groups showed that all patient participants had sufficient initiative and motivation (mean RA > 0.97 in the two patient groups), though the task adopted in this work was relatively simple. The main group effect was revealed in both RT (*H* = 28.342, *p* < 0.001) and RA (*H* = 16.081, *p* < 0.001) in the visual oddball task. Further multiple comparison tests showed that the RT of the HC group was significantly shorter than that of the CS (*p* = 0.006) and SS (*p* < 0.001) groups. Meanwhile, RA was significantly higher in the HC group compared with the CS (*p* = 0.010) and SS (*p* < 0.001) groups. After multiple comparison analysis, no significant difference was observed in the behavioral metrics between the two groups of patients. Considerably worse behavioral performance in both groups of patients may indicate impaired cognitive function involving visual information processing in mild stroke patients.

### 3.2. Differences in EEG Functional Connectivity

**Figure 3:**
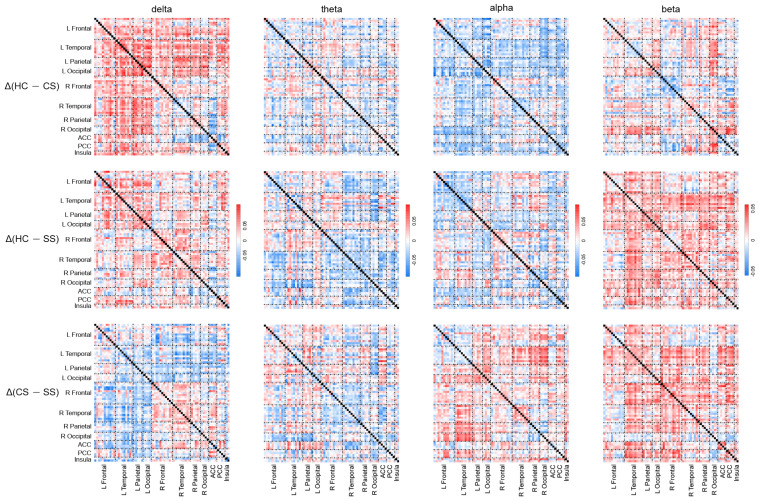
PEC differences between each two groups. The PEC difference was defined as the difference between the mean PEC values of the two groups. Top panel: differences between the HC and CS groups. Middle panel: differences between the HC and SS groups. Bottom panel: differences between the CS and SS groups.

**Figure 4:**
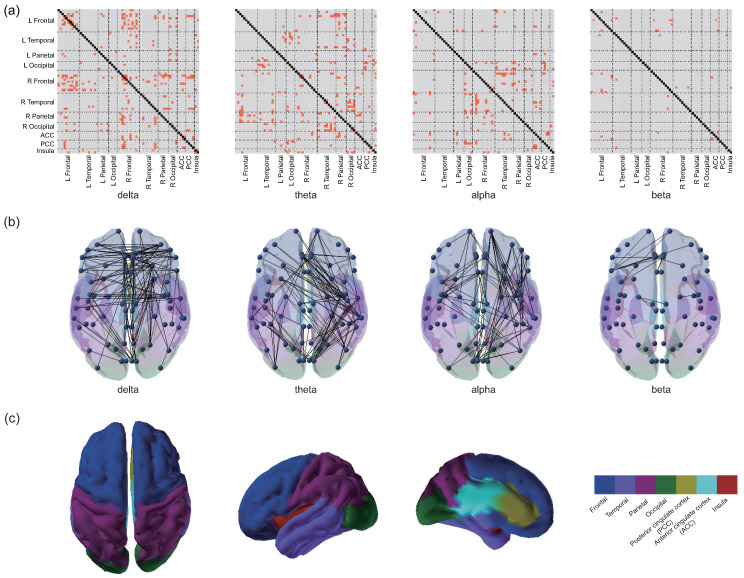
(**a**) Spatial and spectral distribution of significantly different PECs among the three groups (assessed by one-way ANOVA, and Bonferroni corrected). PECs with significant differences (*p* < 0.05) are highlighted in red. According to [45,54], the 68 ROIs were further grouped into 7 ROIs to obtain a clearer distribution of PEC in specific brain regions. L, left; R, right. (**b**) Topographies of significantly different PEC patterns in the brain. (**c**) Illustration of the grouped 7 ROIs (frontal, temporal, parietal, occipital, posterior cingulate cortex (PCC), anterior cingulate cortex (ACC), and insula).

To visualize the comparison of PEC characteristics across the three groups, Figure 3 depicts the PEC difference matrices between each of the two groups in each frequency band. Figure 4 shows the distribution of PECs with statistically significant differences among the three groups, which were assessed by one-way ANOVA. Of note, to obtain a clearer distribution of PEC in the specific brain regions, in the presentation of the statistical results, the parcels were grouped into seven different ROIs, including frontal, temporal, parietal, occipital, posterior cingulate cortex (PCC), anterior cingulate cortex (ACC), and insula [45,54] (Figure 4c). As shown in Figure 4, among the three groups, statistically significant PEC differences occurred mainly in the delta, theta, and alpha frequency bands, with the least distribution in the beta band (visualization of the patterns of connectivity differences among the three groups on the brain is shown in Figure 4b). Specifically, in the delta band, most PECs with significant differences occurred in the frontal cortex. In the theta band, the PECs with significant differences were mainly from the frontoparietal and temporal–occipital regions. In addition, the most frequently occurring PEC differences in the alpha band were located in the frontal–temporal area. In the beta frequency band, although the number of significant PEC differences was the least, they were mainly distributed in the frontal area. Complex distribution patterns in the frequency band and cortex were observed in PECs with significant differences among the three groups, indicating intricate topological changes induced by mild stroke with different lesion locations during cognitive processing.

### 3.3. Analysis of Networks Metrics

**Figure 5:**
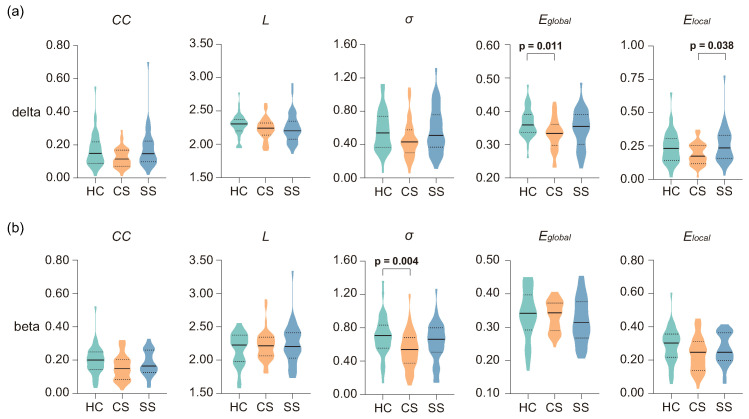
Post hoc statistical analysis of global metrics in the (**a**) delta and (**b**) beta frequency bands. The first and third quartiles are shown as dashed black lines and the median as solid black lines. A *p* value in **bold** represents that the difference is statistically significant (*p* < 0.05).

Table 3 presents the statistical results of the network metrics in four frequency bands. Significant main effects of group were found in the delta and beta bands. Specifically, in the delta frequency band, significant differences in network topological metrics, including CC (*H* = 6.839, *p* = 0.033), $E_{global}$ (*H* = 8.481, *p* = 0.014), and $E_{local}$ (*H* = 6.802, *p* = 0.033), were observed among the three groups. In the beta band, σ showed a significant difference among the three groups (*H* = 10.313, *p* = 0.006). Figure 5 showed further post hoc statistical results for the network metrics in the delta and beta bands. As illustrated in Figure 5a, the analysis revealed a marginally significant effect of CC between the two patient groups (*p* = 0.054), with slightly higher values in the SS group. In terms of efficiency metrics, $E_{global}$ was significantly higher in the HC group in comparison with the CS group (*p* = 0.011), while $E_{local}$ of the CS group was significantly lower than that of the SS group (*p* = 0.038). The post hoc statistical results of the beta band are shown in Figure 5b; the σ of the HC group increased significantly compared with the CS group (*p* = 0.004).

### 3.4. Classification Performance

**Figure 6:**
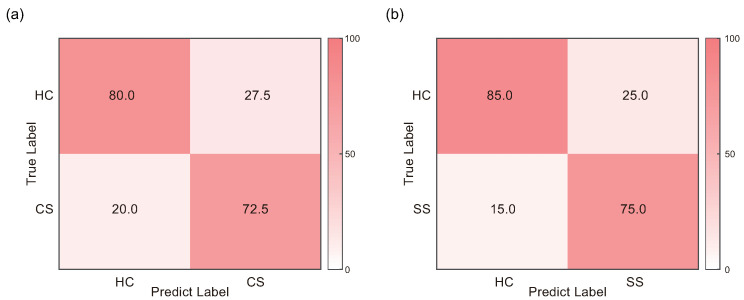
Confusion matrix for best classification performance. (**a**) Confusion matrix for HC and CS classification in the combination of LARS and Boost. (**b**) Confusion matrix for HC and SS classification in the combination of Relief and Tree.

**Figure A1:**
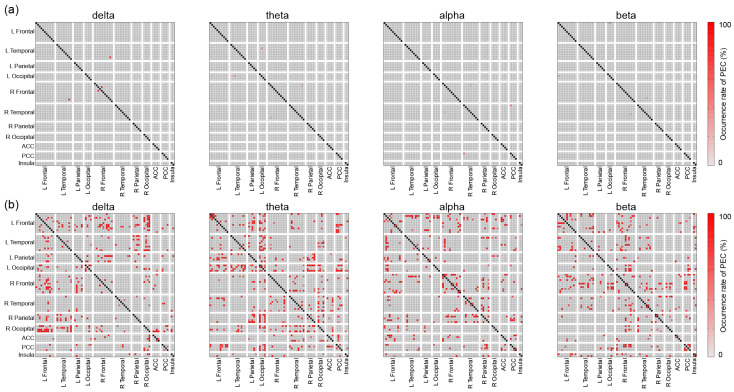
Distribution of selected PEC features in each frequency band. (**a**) Selected features in the classification of HC and CS groups, and (**b**) selected features in the classification of HC and SS groups.

The classification results obtained from various combination of feature selection methods and classifiers are presented in Table 4 and in Table A2. SEM was calculated from the accuracy of each iteration of LOOCV, reflecting the precision of the classification accuracy obtained by LOOCV. The highest classification accuracy (76.25%; sensitivity = 78.38%, specificity = 74.42%, Figure 6a) in discriminating HC and CS was achieved by the combination of LARS and Boost. On the other hand, the best classification performance of HC and SS was obtained by the combination of Relief and Tree, with an accuracy of 80.00% (sensitivity = 83.33%, specificity = 77.27%, Figure 6b). Notably, the optimal number of features for HC and CS classification was 9, while that for HC and SS classification was 757. The selected PEC features are illustrated in Figure A1. Additionally, the classification results of CS and SS groups are further provided in Table A3.

## 4. Discussion

To investigate the influence of mild stroke and lesion location on cognitive function, we assessed task performance as well as the functional brain network characteristics during the visual oddball task. Firstly, we found that behavioral performance was significantly worse in both patient groups. Secondly, PEC patterns across the three groups presented complex brain distribution in each frequency band. Moreover, the reorganization of brain networks was shown in the patient groups, which was reflected in altered information transfer efficiency and reduced small-worldness. Thirdly, the effectiveness of PEC features in classifying mild strokes was demonstrated by further classification analysis. These results are largely consistent with our hypothesis. This is described in more detail as follows.

### 4.1. Worse Task Performance in Patient Groups

As expected, there was a significant decline in task performance in the two patient groups as indicated by the longer RT and lower RA. RT is a measure of information processing speed; a longer RT indicates a delay in the target visual stimuli detection, which was common in stroke patients [55,56]. Meanwhile, a significant reduction in RA was shown in stroke patients in previous studies [56,57]. For instance, Hsu and colleagues recruited patients with putamen and thalamic stroke to perform a visual oddball task to investigate working memory function; significantly worse behavioral performance indicated deficits in visual information processing in stroke patients [57]. RA was relatively high in both patient groups, reflecting, to some extent, the relatively simple task design, which may explain the lack of significant behavioral differences between the two groups. Nonetheless, our findings extend the results of the prior research to mild stroke, which may further support the idea that localized brain damage is strongly associated with behavioral deficits caused by stroke [58,59].

### 4.2. Complex Functional Connectivity Distribution

The delta frequency band is closely associated with signal detection and decision-making functions [60,61], whereas the visual oddball task requires participants to focus on detecting target stimuli and making a decision. The altered EEG functional connectivity in the delta frequency band may reflect differences in cognitive function, including signal detection and decision making, between stroke patients and healthy controls. Similarly, frequency-specific altered functional connectivity pairs were found in a previous study, with stroke patients showing significantly reduced frontoparietal connectivity during a visual oddball task [56]. Furthermore, it should be noted that the frontal cortex plays a key role in attentional function [62], whereas frontal delta activity is related to visual perception [63]. Therefore, the predominant distribution of significant functional connectivity differences located in the frontal region may further suggest altered cognitive functions in the patient groups. Theta oscillation has primarily been reported to be associated with attentional control processes [64], a critical cognitive function during performing the visual oddball task. In addition, the frontoparietal network has been shown to be involved in cognitive performance [65], which is supported by studies of brain lesions and the analysis of cognitive data from healthy controls using various neuroimaging methods [66,67]. Given that cognitive performance can be reflected in behavioral performance, significant differences in task performance among groups may help to explain the main connectivity differences that came from the frontoparietal regions. Meanwhile, the important role of temporal–occipital regions in visual object recognition suggest that cognitive abilities related to the processing of visual information differ between healthy and stroke patients [68]. Alpha band coherence measures conscious processes and provides a state of readiness to engage in cognitive activity [69,70]. This is corroborated by studies showing that abnormal alpha coherence is linked to neurological dysfunction, particularly in stroke patients [71]. As frontotemporal areas are involved in inhibition and executive functions [72], the significantly different alpha band connectivity distributions can be interpreted as differences in cognitive executive function between stroke patients and healthy participants. In addition, frontal beta-band oscillations were shown to be associated with top-down control mechanisms [73], whereas during the visual oddball task, the top-down control involved was attentional control under internal guidance [74]. Significant differences in PECs located in the frontal regions of the beta band suggest differences in attentional function between healthy controls and stroke patients.

### 4.3. Topological Alterations of Brain Network

Efficient neural communication within brain networks is essential for brain function [75]. Functional networks in the human brain typically have high degrees of segregation and integration, promoting local and global information processing efficiency [76]. Specifically, global efficiency, which reflects the efficiency with which brain-wide networks transmit information, is related to cognitive performance and cognitive dysfunction in patients with cerebral small vessel disease [30,77]. Consistent with previous functional brain network studies in post-stroke patients [78,79], a significant reduction in global information flow efficiency was shown in cortical stroke patients. Furthermore, the significant differences in network efficiency metrics were only found in the delta band, which has repeatedly been shown to be linked to signal detection and decision-making functions and to be important for large-scale cortical integration [60,61,80]. We, therefore, speculate that patients with cortical stroke have deficits in cortical integration and impairments in information processing efficiency for target detection and decision making. Notably, patients with cortical and subcortical strokes showed a significant difference in local efficiency. As a measure of functional segregation, local efficiency represents specialized processing in groups of densely interconnected regions of the brain [26]. The results suggest that cognitive dysfunction in patients with cortical stroke may be related to decreased connection density in specialized networks involved in cognition. However, the studies investigating the impact of different lesion locations on cognitive function are still limited and inconsistent. By definition, σ evaluates the balance between the global integration and the local processing of a network, reflecting the optimal network structure associated with rapid synchronization and information transfer [81]. In comparison with healthy controls, rearrangement of the brain network, which is reflected in the reduction in σ, has been found in stroke patients [18,82]. Given that beta-band oscillations are associated with executive function, our findings suggest the reduced efficiency of neural processing during task performance in patients with cortical stroke. Overall, the disruption of global information transmission in patients with cortical stroke indicates that cognitive function was impaired in visual information processing. Meanwhile, although all patients had mild stroke symptoms, cognitive impairment may be more severe in patients with cortical lesions, as local efficiency and small-worldness were significantly reduced in cortical stroke patients compared with subcortical stroke patients and healthy controls, respectively. Our findings provide new evidence for differences in cognitive function between patients with different lesion locations, from the perspective of alterations in functional brain network architectures.

### 4.4. Classification Performance

The feasibility of PECs as features for identifying mild stroke was validated by satisfactory classification performance for classifying HC and CS, and HC and SS, respectively. Notably, patients with moderate to severe stroke were the main focus of previous studies on differentiating stroke patients [83,84,85]. For instance, based on the machine-learning approach, Rahma et al. and Hussain et al. achieved classification accuracies of 72% and 85% by using EEG spectral metrics (i.e., power spectral density (PSD)-related metrics) as features, respectively [83,85]. Additionally, different from prior classification studies of resting-state EEG signals using commonly univariate EEG PSD features [83,84,86], the current work employed multivariate PECs estimated during cognitive tasks as features. In fact, EEG functional connectivity features are suitable for detecting hidden layers of cognitive functional reorganization in the brain, which are able to achieve high classification performance [87,88]. Furthermore, although traditional scales, including the NIHSS, Barthel Index and modified Rankin Scale, are widely used to detect stroke [89], these scales have limited utility in the assessment of cognitive domains and have limited ability to detect small changes in functional status [90,91]. Given that the patients recruited in this study all had mild stroke symptoms and the classification methods adopted were all traditional and universal, the comparable classification performance (accuracy of 76.25% in classifying HC and CS, 80% in classifying HC and SS) further suggests that PECs have potential as biomarkers in identifying patients with mild stroke.

### 4.5. Limitations

In this paper, there are some issues that need to be considered when interpreting our results. First, although we considered different lesion locations (i.e., cortical and subcortical stroke), the inherent heterogeneous characteristics of the lesioned hemisphere may hinder us from drawing clear conclusions. Additionally, the significantly larger number of features than the sample size would introduce overfitting and limit the generalization ability of the classification model. Therefore, future studies with larger sample sizes, including patients with different lesion hemispheres and locations, should be considered. Second, the cognitive task used in the current work was relatively simple in order to maintain task cooperation and motivation in older subjects, which also limited the types of cognitive functions that could be assessed. More and relatively complex cognitive tasks could be used in future studies to obtain more information about other cognitive functions (e.g., working memory, spatial processing, and visuoconstruction). Third, although the classification in the current work was an exploratory analysis to investigate the feasibility of PEC as a feature to identify mild strokes, future studies could further focus on using functional connectivity as input to distinguish mild strokes at different lesion locations. Specifically, from the perspective of input features, directed functional connectivity can be considered due to the advantage of investigating the causal influence of one brain region on another [92]. Recently, a study of Granger causality analysis in patients with subacute stroke showed that changes in effective connectivity between the bilateral hippocampus and cingulate gyrus were associated with cognitive function after stroke [93]. Furthermore, the feasibility of effective connectivity in stroke clinical prognosis prediction was demonstrated [94]. Therefore, we speculate that directed functional connectivity may perform well in mild stroke detection, providing not only connectivity changes but also the direction of information flow between brain regions. From the perspective of classifiers, deep learning methods and fusion methods of features or decisions would be an optional promising approach to improve the classification performance [95,96].

## 5. Conclusions

This work aims to explore the impact of mild stroke (i.e., cortical and subcortical lesion) on cognitive function during cognitive processing, utilizing the EEG source connectivity method. The significantly worse cognitive performance demonstrates impaired cognitive function in visual information processing in mild stroke patients. In addition, PEC with significant differences among the three groups presents complex distribution patterns in the frequency band and brain region, indicating intricate brain network topology alterations induced by different lesion locations. Reduced global efficiency and small-worldness imply deficits in the efficiency of information transfer during cognitive processing in cortical stroke patients. Meanwhile, significantly decreased local efficiency suggests that impaired cognitive function may be more severe in subcortical stroke patients as compared to those with cortical stroke. The classification results demonstrate the feasibility of PECs as features in the classification of mild stroke. To conclude, the findings obtained provide preliminary insights into the neural mechanisms related to impaired cognitive function in patients with mild stroke at different lesion locations, which may help to further explore cognitive rehabilitation in mild stroke.

## Figures and Tables

**Table 1:** Characteristics of the healthy controls and the stroke patients.

	HC (N = 40)	CS (N = 40)	SS (N = 40)	*p*-Value
Gender (M/F)	15/25	22/18	22/18	0.195 ^a^
Age (years)	62.58 ± 0.92	64.93 ± 1.73	62.20 ± 1.68	0.376 ^b^
NIHSS	-	1.74 ± 0.36 ^c^	1.67 ± 0.27 ^d^	0.377 ^e^
Educational attainment (years)	6.83 ± 0.65	7.50 ± 0.69	6.83 ± 0.67	0.647 ^e^
Time after stroke (days)	-	72.05 ± 27.53	64.80 ± 39.14	0.379 ^f^

Values are expressed as mean ± standard error of mean (SEM). ^a^ Chi-square test. ^b^ One-way ANOVA test. ^c^ Missing data for 5 patients in the CS group. ^d^ Missing data for 4 patients in the SS group.^e^ Kruskal–Wallis test. ^f^ Mann–Whitney *U* test.

**Table 2:** Statistical comparison of visual oddball behavioral results using the Kruskal–Wallis test.

	HC	CS	SS	*H*-Value	*p*-Value	Multiple Comparison Test (*p*-Value ^a^)		
	Mean ± SD	Mean ± SD	Mean ± SD			HC/CS	HC/SS	CS/SS
RT	316.754 ± 37.880	366.390 ± 82.805	388.662 ± 76.989	28.342	**<0.001**	**0.006**	**<0.001**	0.087
RA	0.993 ± 0.009	0.976 ± 0.037	0.974 ± 0.034	16.081	**<0.001**	**0.010**	**<0.001**	1.000

RT means reaction time, RA means response accuracy. *H* is the test statistic for the Kruskal–Wallis test. ^a^ means *p*-value corrected by Bonferroni. A *p* value in **bold** represents the difference is statistically significant (*p* < 0.05).

**Table 3:** Kruskal–Wallis test results for network metrics.

	Delta		Theta		Alpha		Beta	
	*H*-Value	*p*-Value	*H*-Value	*p*-Value	*H*-Value	*p*-Value	*H*-Value	*p*-Value
CC	6.839	**0.033**	0.235	0.889	3.019	0.221	5.222	0.073
*L*	3.862	0.145	0.726	0.696	0.199	0.905	0.487	0.784
σ	4.950	0.084	0.505	0.777	0.824	0.662	10.313	**0.006**
$E_{global}$	8.481	**0.014**	1.639	0.441	4.544	0.103	1.952	0.377
$E_{local}$	6.802	**0.033**	0.565	0.754	3.344	0.188	4.987	0.083

*H* is the test statistic for the Kruskal–Wallis test. A *p* value in **bold** represents that the difference is statistically significant (*p* < 0.05).

**Table 4:** Classification results of various combinations of FS methods and classifiers.

Classifiers	HC vs. CS (ACC ± SEM (%))				HC vs. SS (ACC ± SEM (%))			
	Corr	Fisher	Relief	LARS	Corr	Fisher	Relief	LARS
LR	66.25 ± 5.32	67.50 ± 5.27	65.00 ± 5.37	71.25 ± 5.09	65.00 ± 5.37	65.00 ± 5.37	46.25 ± 5.61	55.00 ± 5.60
Boost	63.75 ± 5.41	63.75 ± 5.41	68.75 ± 5.21	**76.25 ± 4.79**	70.00 ± 5.16	70.00 ± 5.16	51.25 ± 5.62	60.00 ± 5.51
Tree	60.00 ± 5.51	60.00 ± 5.51	61.25 ± 5.21	65.00 ± 4.79	63.75 ± 5.41	63.75 ± 5.41	**80.00 ± 4.50**	58.75 ± 5.54
RF	57.50 ± 5.56	58.75 ± 5.54	63.75 ± 5.41	70.00 ± 5.16	55.00 ± 5.60	55.00 ± 5.60	53.75 ± 5.61	57.50 ± 5.56

The highest classification accuracy is indicated by the **bold** font.

## Data Availability

Not applicable.

## Appendix A

### Appendix A.1. Supplementary Materials for Patients with Mild Stroke

Table A1 lists the specific forms of rehabilitation performed on the patients recruited in the current work.

**Table A1:** The specific form of rehabilitation employed by the patients recruited in the current work.

Rehabilitation Form	Number of Patients
Medication	60
Medication; Acupuncture treatment	2
Medication; Exercise therapy	1
Medication; Acupuncture treatment; Exercise therapy; Occupational therapy	6
Medication; Cerebral angiography	2

Note: Missing data for 9 patients.

### Appendix A.2. Supplementary Classification Results

In Table A2, we provide additional information on the sensitivity and specificity of the classification results presented in Table 4. In Figure A1, we provide the distribution of the selected PEC features in the classification of the HC and CS groups, and the HC and SS groups, respectively. Notably, the number of selected features in the HC and CS classification is relatively small. Since LARS solves an L1-norm constrained problem, it inherently produces sparse results, selecting only a few features most relevant to the classification task [97], which may explain the relatively few features selected in the HC and CS group classification.

**Table A2:** The sensitivity and specificity of classification results for various combinations of FS methods and classifiers.

Classifiers	HC vs. CS (ACC (Sensitivity/Specificity) (%))				HC vs. SS (ACC (Sensitivity/Specificity) (%))			
	Corr	Fisher	Relief	LARS	Corr	Fisher	Relief	LARS
LR	66.25 (66.67/65.85)	67.50 (68.42/66.67)	65.00 (63.64/66.67)	71.25 (71.79/70.73)	65.00 (66.67/63.64)	65.00 (66.67/63.64)	46.25 (46.34/46.15)	55.00 (55.26/54.76)
Boost	63.75 (62.79/64.86)	63.75 (62.79/64.86)	68.75 (67.44/70.27)	**76.25 (78.38/74.42)**	70.00 (68.18/72.22)	70.00 (68.18/72.20)	51.25 (52.17/50.88)	60.00 (60.00/60.00)
Tree	60.00 (59.52/60.53)	60.00 (60.00/60.00)	61.25 (61.54/60.98)	65.00 (64.29/65.79)	63.75 (64.10/63.41)	63.75 (64.10/63.41)	**80.00 (83.33/77.27)**	58.75 (58.54/58.97)
RF	57.50 (57.89/57.14)	58.75 (58.97/58.54)	63.75 (64.10/63.41)	70.00 (68.18/72.22)	55.00 (55.00/55.00)	55.00 (55.00/55.00)	53.75 (54.55/53.19)	57.50 (60.00/56.00)

The highest classification accuracy is indicated by the **bold** font.

### Appendix A.3. Classification Results for CS and SS Groups

In Table A3, the classification results between the two patient groups (i.e., CS and SS groups) are shown. The combination of Tree and Corr and Tree and Fisher achieved the highest accuracy of 76.25% (sensitivity = 73.33%, specificity = 80.00%).

**Table A3:** Classification results for CS and SS groups.

Classifiers	ACC (Sensitivity/Specificity) (%)			
	Corr	Fisher	Relief	LARS
LR	67.50 (67.50/67.50)	67.50 (67.50/67.50)	62.50 (62.50/62.50)	55.00 (55.56/54.55)
Boost	63.75 (64.86/62.79)	63.75 (64.86/62.79)	63.75 (64.10/63.41)	63.75 (64.86/62.79)
Tree	**76.25 (73.33/80.00)**	**76.25 (73.33/80.00)**	63.75 (62.22/65.71)	65.00 (64.29/65.79)
RF	67.50 (66.67/68.42)	67.50 (66.67/68.42)	58.75 (58.97/58.54)	57.50 (57.50/57.50)

The highest classification accuracy is indicated by the **bold** font.

### Appendix A.4. 3-Class Classification

We additionally performed a 3-class classification. The previously validated linear support vector machine recursive feature elimination (SVM-RFE) with correlation bias reduction (CBR) was used to filter out redundant and irrelevant PEC features from the original feature dataset and to minimize classification bias due to overfitting [98]. K-nearest neighbors (KNN) was used as the classifier. A classification accuracy of 55% was achieved by the combination of SVM-RFE and KNN, which is above the chance level. The parameter C in SVM training was set to 0.1, and the selected features were 521 PECs.
